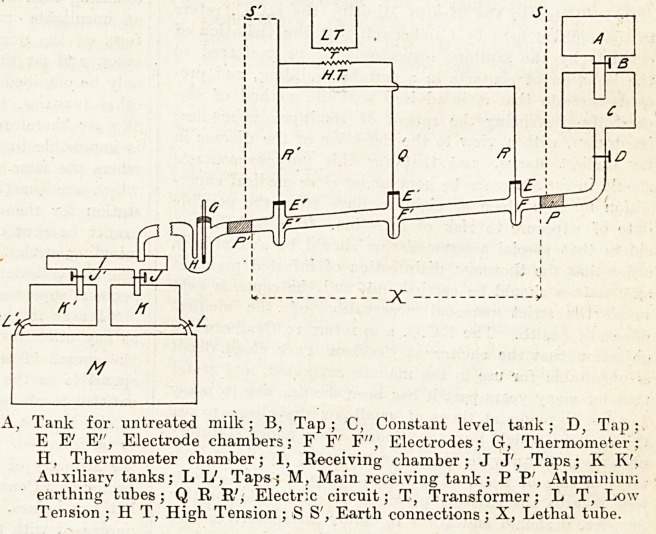# A Recent and Valuable Method

**Published:** 1920-06-05

**Authors:** 


### THE STERILISATION OF MILK BY ELECTRICITY.

### A Recent and Valuable Method.

THOUGH this work of Professor Beattie and Mr. Lewis had already been mentioned in our columns, yet it is one of such promise that a fuller account of the methods employed and the results obtained cannot be considered superfluous.

Before dealing with the results given by the electrical method, it is well to realise the normal condition of milk, and the following table gives the numbers of organisms found in one sample of milk when untreated and when pasteurised by ordinary commercial methods:—

| Sample of | Bacteria per c.c. | *B. coli* per c.c. |
|---|---|---|
| 1. Untreated Milk ... | 3,840,000 | 10,000 |
| 2. Pasteurised (not cooled) | 1,160,000 | nil |
| 3. " (cooled) ... | 240,000 | 10 |

After keeping for three days the numbers of organisms were as follows:—

| Sample of | Bacteria per c.c. | *B. coli* per c.c. |
|---|---|---|
| Untreated Milk ... | 65,600,000 | 100,000 |
| Pasteurised (not cooled) | 168,000,000 | 100,000 |
| " (cooled) ... | 240,000 | 10 |

Such tables suggest that, unless immediately and rapidly cooled, pasteurised milk is as bad, if not worse, than untreated milk. With this unsatisfactory example before us let us now turn to the results obtained by the electrical method.

One instance alone will be enough to show the effects of this method, and below are shown the two comparative readings of milk before treatment, and after:—

| | Untreated | Treated |
|---|---|---|
| Colonies on agar per c.c. | 6,480,000,000 | 980 |
| Colonies on gelatin per c.c. | liquified | 0 |
| Colonies on neutral red lactose agar (*B. coli*) | 720,000,000 | 0 |

This table is of an exceptionally bad specimen, but shows how the organisms fade away under treatment; the readings under the heading "treated" are quite characteristic of those ordinarily obtained.

### THE TECHNIQUE.

The method employed is to allow the milk to flow along a tube, the "lethal tube," into which project three electrodes, the current flowing from the middle electrode to the other two. The diagram below shows the various parts of the apparatus, and the means taken to regulate the supply of milk, and its storage as it leaves the lethal tube.

The factors which were varied in the preliminary experiments were:—(1) The rate of flow of the milk; (2) the terminal voltage; (3) the amount of current used; (4) the temperature of the milk as it left the lethal tube. And the tube efficiency of the process was gauged by the number of organisms surviving per c.c. of milk treated.

At this point it is well to point out that even a momentary failure of current will allow a small quantity of untreated milk to pass, which may infect all the milk previously or subsequently treated. To meet this contingency a special double receiving chamber is used, so that any sample of milk can be trapped and returned for a second treatment should this be desired, and not added directly to the bulk of treated milk.

### VARIOUS RESULTS.

As a result of various experiments it was found that consistently satisfactory results were obtained when the conditions were as follows:—(1) Flow of milk: Two litres in four minutes. (2) Terminal voltage: 2,500 to 3,500 alternating. (3) Amount of current: 0.5 to 0.6 amperes. (4) Temperature of the milk: 62° to 64° Centigrade. Under these conditions all the *B. coli* were destroyed.

A, Tank for untreated milk; B, Tap; C, Constant level tank; D, Tap; E E′ E″, Electrode chambers; F F′ F″, Electrodes; G, Thermometer; H, Thermometer chamber; I, Receiving chamber; J J′, Taps; K K′, Auxiliary tanks; L L′, Taps; M, Main receiving tank; P P′, Aluminium earthing tubes; Q R R′, Electric circuit; T, Transformer; L T, Low Tension; H T, High Tension; S S′, Earth connections; X, Lethal tube.

In a later series of experiments similar satisfactory results were obtained using a lethal tube of four times the cross-sectional area, four times the amount of current, *i.e.*, 2.0 to 2.5 amperes, and four times the flow of milk.

The current necessary for this sterilising effect was obtained by means of an ordinary transformer connected to the ordinary electric supply mains. An alternating current is absolutely essential.

### TUBERCULOSIS.

With regard to *B. coli* the results speak for themselves, but with *B. tuberculosis* a further series of experiments was necessary to prove that

**The Sterilisation of Milk by Electricity—(*continued*).**

the process was lethal to them also. The findings of these experiments were that with the voltage and amperage already noted, if the final temperature of the treated milk reached 63° to 64° Centigrade all tubercle bacilli were killed. Of ten guinea pigs five received the sediment obtained by the centrifugalisation of 500 c.c. of untreated milk, and all developed typical lesions of tuberculosis; five received the sediment of 500 c.c. of the same milk after treatment, and none developed any symptoms of tuberculosis.

The above facts speak for themselves, and show that by employing this method of sterilisation both *B. coli*, and presumably other members of the colon-typhoid group of organisms, and *B. tuberculosis* can be destroyed, and milk rendered perfectly harmless, and that without in any way spoiling its potability. The action of the heat and electricity on the very important accessory food factor "fat soluble A" is not recorded. It is to be hoped that it is not destroyed, for much of the value of this method of treatment is lost should the milk, by the destruction of this vitamine, lose its anti-rachitic properties. This problem has yet to be elucidated.

## Figures and Tables

**Figure f1:**